# Non-Catalytic Domains of DNA Polymerase λ: Influence on Enzyme Activity and Its Regulation

**DOI:** 10.1134/S1607672923700382

**Published:** 2023-12-13

**Authors:** E. A. Maltseva, N. I. Rechkunova, O. I. Lavrik

**Affiliations:** Institute of Chemical Biology and Fundamental Medicine, Siberian Branch, Russian Academy of Sciences, Novosibirsk, Russia

**Keywords:** base excision repair, DNA polymerases, PARP1, activity regulation

## Abstract

DNA polymerase λ (Polλ) belongs to the same structural X-family as DNA polymerase β, the main polymerase of base excision repair. The role of Polλ in this process remains not fully understood. A significant difference between the two DNA polymerases is the presence of an extended non-catalytic N-terminal region in the Polλ structure. The influence of this region on the interaction of Polλ with DNA and multifunctional proteins, poly(ADP-ribose)polymerase 1 (PARP1) and replication protein A (RPA), was studied in detail for the first time. The data obtained suggest that non-catalytic Polλ domains play a suppressor role both in relation to the polymerase activity of the enzyme and in interaction with DNA and PARP1.

DNA polymerase λ (Polλ) belongs to the same structural X-family as DNA polymerase β (Polβ), the main polymerase in the base excision repair (BER) system. Both enzymes possess activities required for reparative synthesis: they catalyze DNA synthesis in gaps, including those on damaged templates, exhibit 5'-dRP-lyase activity, and are also able to perform strand displacement synthesis [1, 2]. Polβ is recognized as the key enzyme in the process of filling the single-nucleotide gap that occurs during BER, whereas the role of Polλ in this process remains not fully understood.

DNA damage in BER is repaired in several stages, at each of which specific DNA intermediates are formed under the action of certain enzymes. An important role in the process is played by the coordination of enzyme functions at each stage due to interactions with partner proteins that regulate enzyme activity [3]. One of the key protein regulators of DNA repair, poly(ADP-ribose) polymerase 1 (PARP1), interacts with damaged DNA and catalyzes the synthesis of the polymer ADP-ribose using NAD^+^ as a substrate. We have previously shown that PARP1 modulates Polβ activity on DNA intermediates of the short- and long-patch BER pathways [4]. On the other hand, in the presence of DNA structures containing a single-nucleotide gap or a nick, which model BER intermediates at the stage of DNA resynthesis, PARP1 activity is stimulated by the replication protein A (RPA) [5]. It was also shown that RPA stimulates Polλ activity, in particular, during synthesis on a damaged template [6]. Both proteins, PARP1 and RPA, are present in the cell in significant amounts and are involved in many processes. For this reason, we studied their influence on the activity of Polβ and Polλ in order to determine whether the observed effect of PARP1 activity stimulation in the presence of RPA can manifest itself during repair synthesis.

We performed a comparative analysis of the effects of PARP1 and poly(ADP-ribosyl)ylation on the activity of Polβ and Polλ in the short- and long-patch BER pathways. Radioactively labeled DNA duplex containing a single-nucleotide gap with 5′-phosphate (gap1-p-DNA) was incubated with DNA polymerase and dNTP at various concentrations, at which one nucleotide was inserted or strand displacement DNA synthesis was observed (Fig. 1). Reactions in the presence of PARP1 and/or RPA in the absence or presence of NAD^+^ were performed similarly. The analysis showed that PARP1 inhibited the activity of both DNA polymerases; however, the inhibitory effect depended both on the DNA polymerase and on its concentration.

**Fig. 1.**
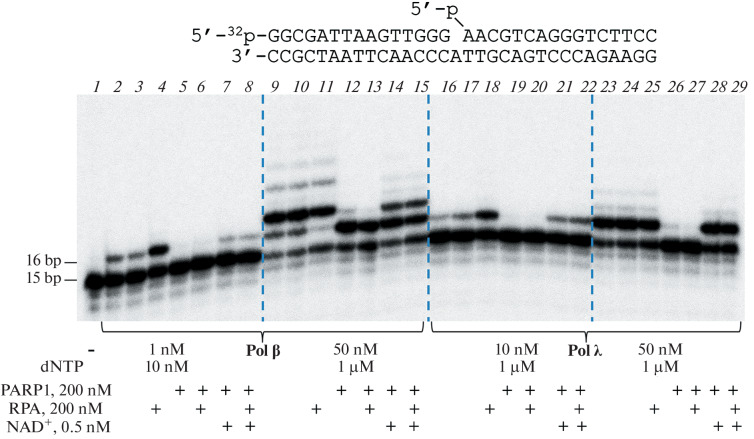
Effect of PARP1, poly(ADP-ribose) synthesis, RPA, and the co-presence of PARP1 and RPA on the activity of DNA polymerases β and λ in the reactions of single-nucleotide gap filling and strand displacement synthesis. Analysis of reaction products by PAGE in denaturing 10% polyacrylamide gel. A radioactively labeled (at the 5'-end of the primer) DNA duplex (20 nM) containing a single-nucleotide gap with 5'-phosphate (gap1-p-DNA) was incubated with DNA polymerase and dNTP at the indicated concentrations at 25°C for 30 min in the presence of PARP1 and/or RPA in the absence or presence of NAD^+^ (as indicated in Fig. 1). All reaction mixtures contained 50 mM Tris-HCl (pH 7.5, 25°C), 50 mM NaCl, 5 mM MgCl_2_, and 50 μg/mL BSA.

For Polβ, under conditions of single-nucleotide gap filling, an almost complete reaction inhibition was observed (Fig. 1, lane *5*), whereas at a higher concentration of DNA polymerase, the PARP1 protein suppressed only the strand displacement synthesis but had almost no effect on the single-nucleotide gap filling (Fig. 1, lane *12*). In the case of Polλ, an almost complete inhibition of not only the strand displacement synthesis but also the single-nucleotide gap filling was observed (Fig. 1, lanes *19* and *26*). It should be noted that Polλ was significantly less efficient in the strand displacement synthesis reaction than Polβ (Fig. 1, cf. lanes *9*, *10* and *23*, *24*), as was shown earlier [7, 8]. In the presence of NAD^+^, the inhibitory effect of PARP1 was significantly reduced, especially in the case of the gap filling reaction catalyzed by Polλ (Fig. 1, lane *21*). The addition of RPA to the reaction mixtures also had different effects: at a low concentration of DNA polymerases, RPA stimulated their activity (Fig. 1, lanes *4* and *18*), whereas at a high concentration, it inhibited them, largely through the effect on the single-nucleotide gap filling than on the strand displacement synthesis (Fig. 1, lanes *11* and *25*). In the case of combined addition of PARP1 and RPA, even under conditions of single-nucleotide gap filling, RPA not only did not remove the inhibitory effect of PARP1 but even moderately enhanced it, both in the absence (Fig. 1, lanes *6*, *13*, *20*, *27*) and in presence (Fig. 1, lanes *8*, *15*, *22*, *29*) of NAD^+^. Thus, the activity of Polβ and Polλ can be modulated by PARP1 and RPA proteins depending on the ratio of the concentrations of these proteins. However, the effect of RPA-dependent stimulation of PARP1 on the activity of DNA polymerases did not manifest itself explicitly in our experiments.

The main difference between the studied DNA polymerases is the presence of an extended non-catalytic N-terminal region, which includes the BRCT domain and the serine/proline-rich region, in the Polλ structure (Fig. 2a). On the one hand, these domains affect the catalytic activity of the enzyme; on the other hand, they can be involved in protein–protein interactions with other repair factors and undergo posttranslational modifications [9–11]. Possibly, the difference in the effect of PARP1 on the activities of Polβ and Polλ is determined, among other things, by the interaction of this protein with the non-catalytic site of Polλ. With this in mind, we obtained the mutant form of Polλ∆N containing only the catalytic β-like domain (244 aa were removed from the N terminus) [12, 13]. It should be noted that the removal of the non-catalytic site led to an increase in the enzyme activity in the gap filling reaction in gap1-p-DNA almost to the level of Polβ (Fig. 2, cf. panels (b) and (d)). The efficiency of strand displacement synthesis catalyzed by Polλ∆N also significantly increased compared to the full-length Polλ. As can be seen from the diagrams presented in Fig. 2, the effect of PARP1 and RPA on the activity of Polλ∆N largely coincided with the effect of these proteins on Polβ than on Polλ. Thus, the non-catalytic Polλ domains affect not only the activity of the enzyme but also the regulation of this activity.

**Fig. 2.**
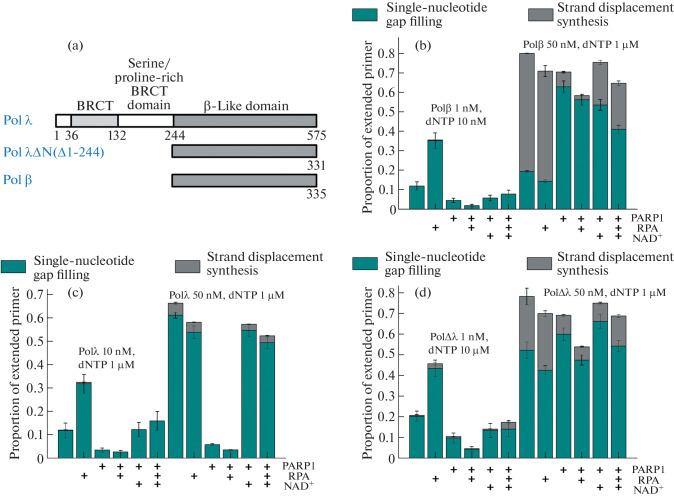
Effect of PARP1, poly(ADP-ribose) synthesis, RPA, and co-presence of PARP1 and RPA on the activity of DNA polymerases β, λ, and λΔN in the reactions of single-nucleotide gap filling and strand displacement synthesis. (a) Schematic representation of the domain structure of Polβ, Polλ, and Polλ∆N. BRCT domain—C-terminal BRCA1 domain. (b–d) Proportion of extended primer determined as the ratio of the intensity of the reaction products to the intensity of the initial primer for the reaction catalyzed by Polβ, Polλ and PolλΔN, respectively. Radioactively labeled (at the 5' end of the primer) DNA duplex (20 nM) containing a single-nucleotide gap with 5′-phosphate (gap1-p-DNA) was incubated with DNA polymerase and dNTP at the indicated concentrations at 25°C for 30 min in the presence of 200 nM PARP1 and/or 200 nM RPA in the absence or presence of 0.5 mM NAD^+^ (as indicated in Fig. 2). All reaction mixtures contained 50 mM Tris-HCl (pH 7.5, 25°C), 50 mM NaCl, 5 mM MgCl_2_, and 50 μg/mL BSA.

Next, we compared the efficiency of poly(ADP-ribosyl)ation of the studied DNA polymerases in the presence of gap1-p-DNA. It was found that Polλ is a less effective target for modification than Polβ (we monitored the decrease in the amount of unmodified DNA polymerase with increasing NAD^+^ concentration). The removal of the non-catalytic site led to an increase in the efficiency of modification: the level of Polλ∆N modification practically does not differ from the level of Polβ modification (Fig. 3). Attachment of a negatively charged poly(ADP-ribose) significantly weakens the binding of proteins to DNA, reducing the effective concentration of DNA polymerases. It can be assumed that this effect leads to a significant decrease in the efficiency of strand displacement synthesis for all DNA polymerases under the conditions of poly(ADP-ribose) synthesis.

**Fig. 3.**
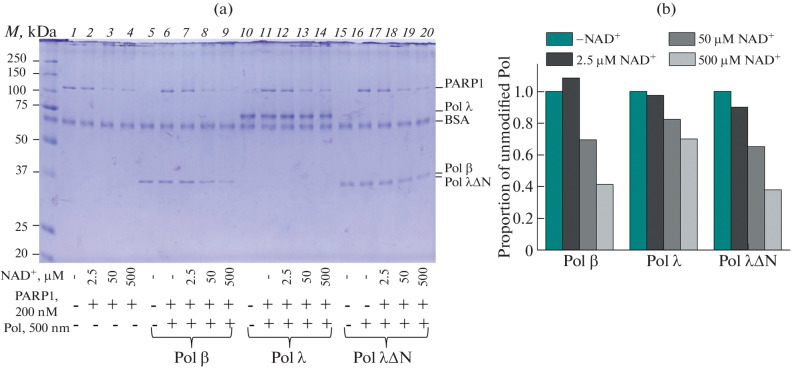
Comparison of the efficiency of poly(ADP-ribosyl)ation of DNA polymerases β, λ, and λ∆N. Analysis of poly(ADP-ribosyl)ation products of Polβ, Polλ, and Polλ∆N by SDS-PAGE in denaturing 12.5% polyacrylamide gel after staining with Coomassie Brilliant Blue R250 (a) and dependence of the proportion of unmodified DNA polymerases (ratio of the intensity of bands in the gel (a) corresponding to the unmodified DNA polymerase in the presence and absence of NAD^+^) on NAD^+^ concentration (b). 200 nM PARP1 was incubated with 500 nM DNA polymerase in the presence of 20 nM DNA duplex containing a single-nucleotide gap with 5'-phosphate (gap1-p-DNA) and 1 μM dNTP at 25°C for 30 min in the absence or presence of NAD^+^ at the indicated concentrations (as indicated in Fig. 3). All reaction mixtures contained 50 mM Tris-HCl (pH 7.5, 25°C), 50 mM NaCl, 50 μg/mL BSA, and 5 mM MgCl_2_.

Differences in the effect of PARP1 on DNA polymerases may also be due to protein–protein interactions. The interaction between Polβ and PARP1 was shown previously [4, 14, 15], whereas such studies for Polλ have not been carried out. It was assumed that the presence of BRCT domains in the structures of Polλ and PARP1 may significantly contribute to the interaction between these proteins. A preparation of PARP1 fluorescently labeled at the N-terminal amino group (Cy3-PARP1) was obtained, and the interaction of this protein with DNA polymerases was evaluated by fluorescent titration. The results of measurements showed that the efficiencies of the interaction of Polβ and Polλ∆N with PARP1 were comparable and almost twice higher compared to the full-length Polλ (Fig. 4). On the one hand, this is consistent with the efficiency of poly(ADP-ribosyl)ation of DNA-polymerases; on the other hand, this suggests a possible interactions between PARP1 and DNA polymerases on DNA. However, the observed difference is still insufficient to unequivocally conclude whether the non-catalytic Polλ region is involved in the binding to PARP1 or whether the binding is due predominantly to the β-like domain. In addition, it should be borne in mind that Polβ and Polλ∆N are not completely identical, and their catalytic domains may interact differently with PARP1 and other repair factors.

**Fig. 4.**
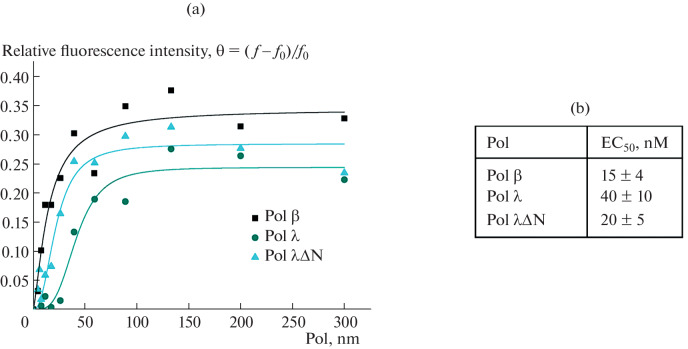
Comparison of the efficiency of PARP1 binding to DNA polymerases β, λ, and λ∆N. Titration curves of fluorescently labeled Cy3-PARP1 (10 nM) with Polβ, Polλ, and Polλ∆N at increasing concentrations (a) and EC_50_ values for PARP1 complexes with DNA polymerases calculated using the Hill equation (b). Hill Equation: $\theta = {{\theta }_{\infty }}\left[ {{\text{1}} + {{{{\text{(E}}{{{\text{C}}}_{{{\text{50}}}}}{\text{/C)}}}}^{n}}} \right]$, where θ is the relative fluorescence intensity of PARP1 in the complex with DNA polymerase at concentration C; θ_∞_ is the maximum relative intensity of PARP1 fluorescence in the complex with DNA polymerase (at saturating concentrations); EC_50_ is the concentration of DNA polymerase at which θ = θ_∞_/2; and *n* is the Hill coefficient (the slope of the curve). Relative fluorescence intensity θ = (*f* – *f*_0_)/*f*_0_, where *f* and *f*_0_ are PARP1 fluorescence intensities in the presence and absence of DNA polymerase, respectively. All reaction mixtures contained 25 mM Tris-HCl (pH 7.5, 25°C), 50 mM NaCl, and 0.5 mM DTT.

Summarizing the obtained data, we assume that, in the absence of NAD^+^, the inhibitory effect of PARP1 on the activity of Polβ and Polλ is determined primarily by the competition for DNA. At the same time, due to protein–protein interactions with PARP1, DNA polymerases can be retained on the DNA substrate, which reduces the efficiency of inhibition. When NAD^+^ is added, PARP1 undergoes autopoly(ADP-ribosyl)ation and releases DNA, which leads to an almost complete restoration of the activity of DNA polymerases. The residual inhibitory effect is explained by poly(ADP-ribosyl)ation of DNA polymerases, which weakens the binding of DNA polymerases to DNA. The effect of PARP1 on the activity of Polβ and Polλ is fundamentally similar but differs in the strength of the effect. This difference is apparently due to the presence of an extended non-catalytic N-terminal region in the Polλ structure. According to the literature, this site is not directly involved in DNA binding [12], but it affects the affinity of the full-length DNA polymerase for DNA. For instance, it was previously shown that the affinity of Polλ for a single-nucleotide gap is 3 times lower than that of Polβ (90 and 30 nM, respectively) [16]. In addition, the presence of this site inhibits protein–protein interactions between Polλ and PARP1. As a result, Polλ is more “sensitive” to the influence of PARP1 than Polβ, since it less effectively competes with PARP1 for a DNA substrate and is less efficiently retained on DNA due to interaction with PARP1.

Thus, the non-catalytic Polλ domains play a suppressor role with respect to both the polymerase activity of the enzyme and the interaction with DNA and PARP1. This may be one of the causes for the key role of Polβ in BER compared to Polλ [17].
